# Achieving Long-Cycle-Life Zinc-Ion Batteries through a Zincophilic Prussian Blue Analogue Interphase

**DOI:** 10.3390/molecules29071501

**Published:** 2024-03-27

**Authors:** Kun Chang, Shuangying Zhao, Wenzhuo Deng

**Affiliations:** 1 College of Chemistry, Fuzhou University, Fuzhou 350108, China; changkun@fjirsm.ac.cn (K.C.); zhaoshuangying@fjirsm.ac.cn (S.Z.); 2 CAS Key Laboratory of Design and Assembly of Functional Nanostructures, Fujian Key Laboratory of Nanomaterials, and State Key Laboratory of Structural Chemistry, Fujian Institute of Research on the Structure of Matter, Chinese Academy of Sciences, Fuzhou 350002, China; 3 Fujian College, University of Chinese Academy of Sciences, Fuzhou 350002, China

**Keywords:** Prussian blue analogues, artificial SEI layer, Zn anode, aqueous zinc-ion batteries

## Abstract

The practical application of rechargeable aqueous zinc-ion batteries (ZIBs) has been severely hindered by detrimental dendrite growth, uncontrollable hydrogen evolution, and unfavorable side reactions occurring at the Zn metal anode. Here, we applied a Prussian blue analogue (PBA) material K_2_Zn_3_(Fe(CN)_6_)_2_ as an artificial solid electrolyte interphase (SEI), by which the plentiful -C≡N- ligands at the surface and the large channels in the open framework structure can operate as a highly zincophilic moderator and ion sieve, inducing fast and uniform nucleation and deposition of Zn. Additionally, the dense interface effectively prevents water molecules from approaching the Zn surface, thereby inhibiting the hydrogen-evolution-resultant side reactions and corrosion. The highly reversible Zn plating/stripping is evidenced by an elevated Coulombic efficiency of 99.87% over 600 cycles in a Zn/Cu cell and a prolonged lifetime of 860 h at 5 mA cm^−2^, 2 mAh cm^−2^ in a Zn/Zn symmetric cell. Furthermore, the PBA-coated Zn anode ensures the excellent rate and cycling performance of an α-MnO_2_/Zn full cell. This work provides a simple and effective solution for the improvement of the Zn anode, advancing the commercialization of aqueous ZIBs.

## 1. Introduction

Zinc (Zn) metal has been considered an ideal anode material for aqueous batteries due to its inherent advantages, such as low cost, high abundance, high theoretical volume capacity (5851 mAh cm^−3^), and a relatively low electrochemical potential versus standard hydron electrode (SHE, −0.76 eV) [1,2,3]. However, the short-circuit problem of zinc-ion batteries (ZIBs) limits their practical application [4]. Hydrogen evolution reactions (HER) frequently accompany the formation of by-products, diminishing the Coulombic efficiency (CE) [5]. Moreover, Zn corrosion and passivation could increase the concentration of OH^-^ ions left behind by water splitting, which will further lower the cycling performance. The development of dendrite-free Zn anodes, which enable highly reversible Zn plating/stripping, is essential to the future success of aqueous Zn-based batteries [6,7,8].

Recently, many novel efforts have been made to solve these problems, and a variety of methods have been proposed to improve Zn anodes’ performance, such as constructing 3D porous framework hosts [9,10], artificial protective films [11,12], electrolyte additives [13,14], and separator modifications [15,16]. However, structural modification of the Zn substrate often requires complex experimental procedures, which limit their application, and the introduction of organic matter sometimes increases the cost and causes environmental problems [17]. Therefore, it remains a huge challenge to reinvent environmentally friendly and easily operated systems for highly durable ZIBs.

To realize highly reversible Zn plating/stripping with dendritic-free morphology, metal-organic frameworks (MOFs) have been used as functional materials to control the diffusion, nucleation, and deposition behaviors of Zn [18,19,20,21,22,23,24]. For example, Liang [22] constructed a homogeneous and stable ZIF-8 layer using controllable electrodeposition. ZIF-8 coating can aid the even deposition and inhibit the corrosion of the Zn sheet surface. Prussian blue analogues (PBAs), as a typical MOF material, display several advantages over the others. The open structure of PBAs offers many three-dimensional (3D) diffusion pathways for the movement of different charge carriers [25,26,27,28,29]. In the past decades, research on PBAs has mainly focused on the electrode materials for application in secondary batteries [30,31,32,33,34]. However, the 3D framework structure in which ion transport can be realized, as well as the rich -C≡N- species in the structure, can also be used as a Zn anode modification strategy. Although Sun et al. [35] and Liu et al. [36] have employed PBA coatings to stabilize the Zn anode, discrepant conclusions were drawn in terms of the hydrophilicity. Therefore, additional work needs to be done to uncover the protection mechanism of the auxiliary PBA layer in aqueous ZIBs.

Here, the shielding of the Zn metal anode by the means of an in-situ PBA coating grown directly on commercial Zn foil has been proposed. The PBA membrane can successfully uniform Zn^2+^-ion flux and modulate the plating morphology at the surface of the Zn anode. In particular, the PBA layer is beneficial for ordered ion transportation and even the nucleation process, thus effectively reducing the dendrite growth. Additionally, the PBA film could serve as a physical interface to prevent direct contact between water and the Zn anode, consequently mitigating HER and increasing the CE. We also computed the binding energy between the H_2_O molecule and the PBA-(002) slab; it is clear that water is more easily trapped at the PBA surface (−1.03 eV) than the bare Zn (−0.29 eV), reflecting the hydrophilic property of the PBA film. The Zn/Zn cell can achieve a stable cycle of more than 860 h at 5 mA cm^−2^, 2 mAh cm^−2^. The assembled full cell using α-MnO_2_ cathode can deliver a long cycle life and remarkably high-rate cycling stability. Both theoretical calculations and experimental characterizations were applied to unveil the mechanism underlying the excellent electrochemical performance.

## 2. Results and Discussion

### 2.1. Characterizations

**Figure 1:**
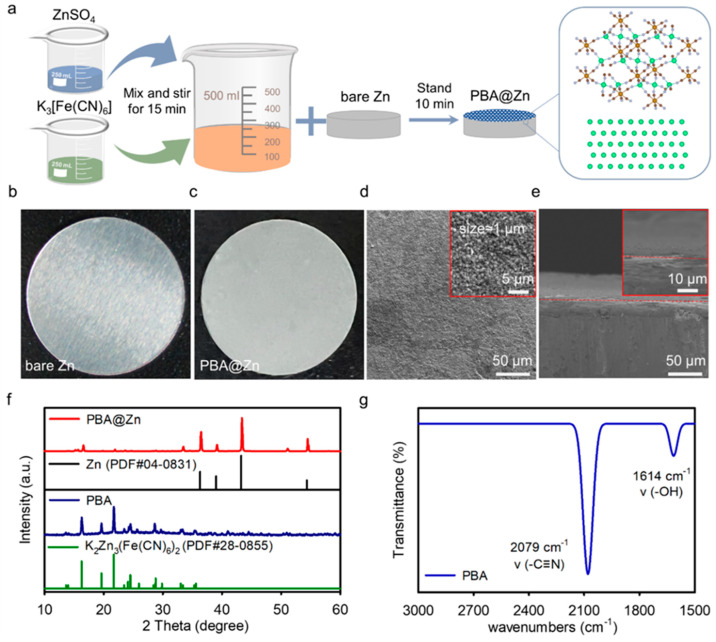
(**a**) The synthesis path and on-site deposition of PBA on the Zn foil, (**b**) The optical image of bare Zn, (**c**) The optical image of the Zn covered with a PBA layer, (**d**) The top and (**e**) cross-sectional SEM images of the PBA@Zn, (**f**) XRD patterns of bare Zn, PBA@Zn, and PBA, (**g**) FT-IR spectrum of PBA layer.

Figure 1a schematically illustrates the process of in-situ growth of the PBA layer on the Zn anode at ambient temperature. We tried different reaction times. The one-minute reaction brought sparse PBA particles to the Zn surface. When the deposition time was prolonged to 5 min, tiny grains gradually grew and clustered. However, the location of the defect can still be observed on the surface (Figure S1). Figure 1b,c is an optical comparison between the bare Zn and the PBA-covered Zn with a deposition time of 10 min. Scanning electron microscope (SEM) (Figure 1d) images demonstrate that a homogeneous and compact PBA coating grew successfully on the Zn surface. The cross-sectional SEM images (Figure 1e) evidenced that a dense and uniform PBA layer was successfully synthesized on the Zn sheet. When the deposition time reaches 20 min, the coating covering the Zn foil is too thick to easily fall off the surface. As shown in Figure S1, small cracks are discernible in the SEM images. Therefore, the deposition time was finally set at 10 min. The X-ray diffraction (XRD) results in Figure 1g show that the diffraction peaks are well-matched to the hexacyanoferrate backbone. The energy-dispersive X-ray spectroscopy (EDX) mapping detects the existence of K, Zn, Fe, N, C, and Zn elements in the PBA-coated Zn (Figure S2), indicating the uniform distribution of the composition of the PBA layer on the surface of the Zn foil. The stretching vibrations of the -C≡N- bonds in the [Fe(CN)_6_]^3−^ groups and the bending vibrations of H–O–H are the two distinctive peaks seen in the Fourier-transform infrared (FTIR) spectra at 2079 and 1614 cm^−1^, respectively (Figure 1g and Figure S3). In summary, the above results confirm that the material was successfully synthesized on the Zn sheet.

### 2.2. Electrochemical Characterizations

**Figure 2:**
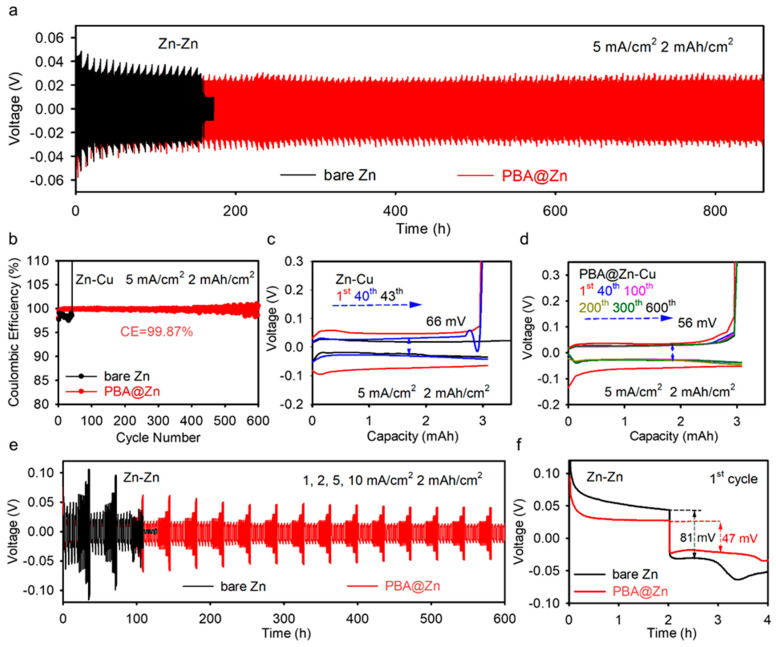
(**a**) Cycling performance of bare Zn and PBA@Zn at 5 mA cm^−2^, 2 mAh cm^−2^ in symmetric cells, (**b**) Coulombic efficiencies of Zn/Cu with or without coating at 5 mA cm^−2^ and 2 mAh cm^−2^, (**c**,**d**) Voltage profiles of Zn/Cu cells with or without coating at 5 mA cm^−2^, 2 mAh cm^−2^, (**e**,**f**) Rate performance at 1, 2, 5, and 10 mA cm^−2^, 2 mAh cm^−2^ in symmetric cells.

Impressively, the symmetric cell using PBA@Zn foil exhibits a stable cyclic life over 860 h at 5 mA cm^−2^, whereas the Zn/Zn cell was short-circuited due to dendrite penetration of the glass fiber separator after a 180 h cycle. Zn/Cu asymmetric cells are used to measure CE to test the protection of the coating at the anode interface. Due to the uncontrolled growth of dendrites on bare Zn, the Zn/Cu cell short-circuited after 43 cycles, while the PBA@Zn/Cu cell could complete over 600 cycles with a high average CE of 99.87% (Figure 2b). The improvement in CE is attributed to the inhibition of side reactions by the shield coating, and the extended cycle life is a result of abundant Zn nucleation sites provided by the PBA layer, which is conducive to the uniform deposition of Zn. Additionally, the PBA coating effectively reduced polarization voltages (Figure 2c,d), demonstrating the homogeneous Zn plating/stripping and inhibited interfacial side reactions. Further examination demonstrates that the membrane could provide excellent rate capability (Figure 2e,f). The symmetric cell with the auxiliary PBA membrane cycled over 600 h after charging and discharging at different current densities. This clearly demonstrates that the coating can enhance the deposition/stripping of Zn at different current densities while maintaining the structural stability and play an important role in improving the cycle life of the battery.

### 2.3. Mechanism

**Figure 3:**
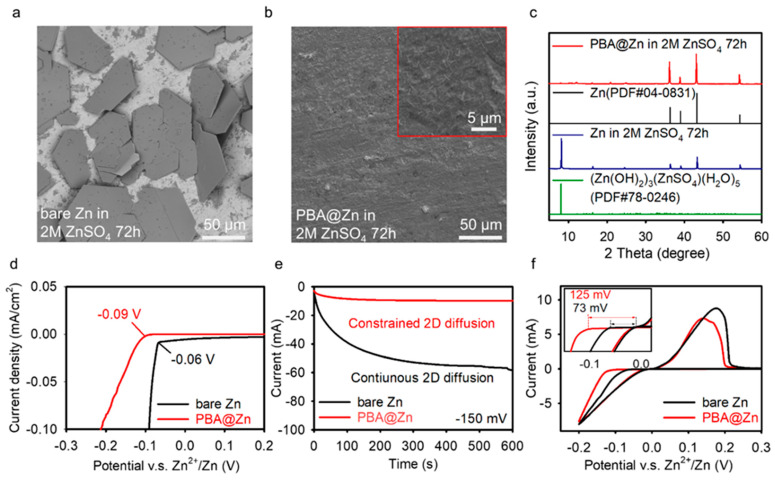
(**a**) The SEM image of bare Zn soaked in 2M ZnSO_4_ for 72 h, (**b**) The SEM images of PBA@Zn soaked in 2M ZnSO_4_ for 72 h, (**c**) XRD patterns of bare Zn foil and PBA@Zn after corrosion in 2M ZnSO_4_ for 72 h, (**d**) LSV characterization of Zn/Ti cells, (**e**) CA characterization of Zn/Zn cells, (**f**) The initial nucleation overpotential of Zn/stainless steel cells.

The anti-corrosion properties of bare Zn and PBA@Zn were studied by soaking in the 2 M ZnSO_4_ electrolyte for 3 days (Figure 3a–c). In contrast to the bare Zn, whose surface was covered with a large amount of (Zn(OH)_2_)_3_(ZnSO_4_)(H_2_O)_5_ films, no significant changes were observed on the PBA@Zn foil, reflecting the excellent corrosion-resistant ability of the PBA layer. PBA@Zn exhibited a lower hydrogen evolution potential, which means that it is more difficult for hydrogen evolution to occur at the PBA-covered Zn surface. (Figure 3d). Zn nucleation and growth behavior were demonstrated by chromatic amperometry (CA) investigations (Figure 3e). The ability to achieve rapid 3D diffusion is closely related to the uniform deposition of zinc. For PBA@Zn, the current reaches a steady state in a very short period of 25 s at an overpotential of −150 mV, indicating that a stable 3D diffusion process has been achieved. Conversely, the current of bare Zn increases gradually throughout 300 s, implying an uncontrollable 2D diffusion process and cumulative Zn deposition. Therefore, the regulated Zn^2+^-ion flux is essential for inducing even Zn nucleation/deposition. This is proved by the surface morphology of the Zn sheet after cycling. We used bare Zn and PBA@Zn to assemble corresponding symmetrical batteries and observed the morphologies after a 50 h cycle at 5 mA cm^−2^, 2 mAh cm^−2^. As shown in Figure S4, large pieces of Zn flakes can be viewed on the top of the cycled bare Zn foil, and some fragments are embedded inside the glass fiber separator. On the other hand, the PBA coating is kept intact, with only a very small amount of Zn deposits and glass fibers present on the surface. In Figure S5, the uneven Zn deposition is also obvious, and some of the metallic pieces are entangled with glass fibers, which may cause short circuit and battery failure. Conversely, only a limited amount of fiber remained on the relatively flat surface of the PBA-coated Zn foil. The XRD results in Figure S6 show apparent diffraction peaks of byproducts from the cycled bare Zn, whereas those peaks are not significant for the PBA-covered Zn foil. There is a minimum critical nucleation radius *r_crit_* for the nucleation of crystals. The *r_crit_* and nucleation overpotential has the following relationship:

(1) $$r_{crit}=\frac{{\gamma V}_{m}}{F|\eta |}$$

where *γ* is the surface energy, *V_m_* is the molar volume, *F* is the Faraday constant, and *η* is the nucleation overpotential. With the increase of *η*, the radius of the crystal nucleus decreases and it is easier to stabilize the fine Zn nuclei. Due to the strong interaction between the coating and Zn^2+^-ion, the abundant zincophilic sites at the PBA surface enable Zn^2+^-ions to migrate rapidly. The ordered nanochannels for uniform Zn^2+^-ion flux provided by the 3D open framework of the PBA interphase and the accelerated desolvation are conducive to the formation of a dense and flat Zn layer instead of loosely packed Zn flakes. As indicated in Figure 3f, the initial nucleation overpotential of bare Zn is 73 mV, which is higher than PBA@Zn (125 mV), suggesting that more nucleation sites are supplied.

The formation of crystals in melts, solutions, or gas phases is a complex and intricate process involving the transformation of atoms from a disordered and chaotic state to an ordered and regular arrangement. The sophisticated procedure is associated with not only simple atomic migration but also precise adjustments in energy transfer, atomic transportation, and interatomic interactions [37]. During the transition from the disordered state to the ordered one, there exists a critical stage suggested by sharp changes in the interface and the newly formed phase, known as the transition zone. Cahn et al. [38] proposed a diffusive interface model, in which the interfacial structure is influenced by the concentration gradient and dynamical atomic diffusion. The path of metal crystallization during electroplating includes several steps. Firstly, metal ions in the solution are converted into atoms and form a transition layer at the interface between the electrode and the electrolyte after capturing electrons. Secondly, the acceleration of atomic hopping and the resulting dimer intermediates promote the formation of atomic clusters. Finally, crystal nuclei are generated through the aggregation of atomic clusters and then grow into large crystals. It is worth noting that, although dimers and atomic clusters are also mobile and can drift around during the deposition process, their diffusion rates are much slower than those of individual atoms. In other words, the atomic diffusion at the surface plays a crucial role in regulating the apparent morphology and properties of the formed crystals, and it not only determines the rate of crystal growth but also influences the shape and quality of the final crystals. Thus, based on previous CA tests and initial nucleation overpotential measurement, it has been observed that the PBA coating has a positive impact on the diffusion of Zn ions and the nucleation routine. This further validates the significance of the coating in the electroplating process.

**Figure 4:**
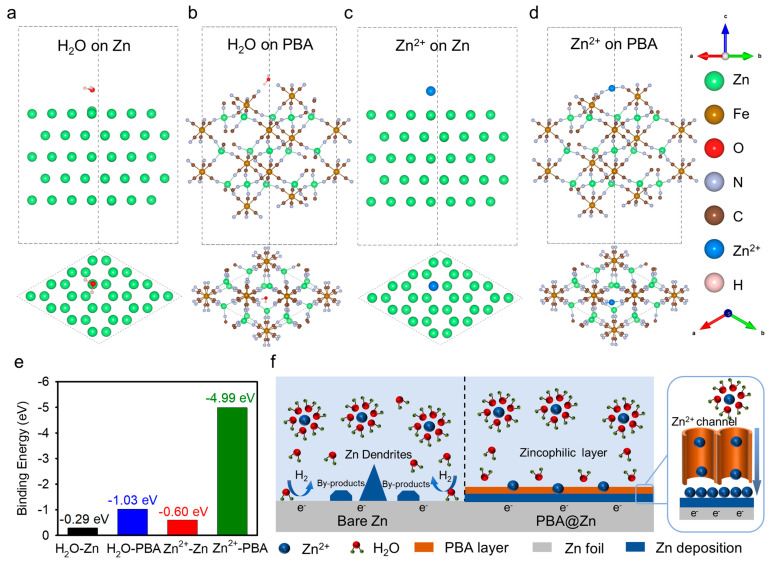
(**a**–**d**) The optimized H_2_O and Zn^2+^ adsorbed configurations, (**e**) Binding energies of Zn^2+^ and H_2_O on the Zn-(002) and PBA-(002) plane, (**f**) Schematic Zn^2+^ deposition on PBA@Zn electrode.

To understand the role of the coating theoretically, we analyzed the interactions between the surface and the naked Zn^2+^-ion. The density functional theory (DFT) calculations (Figure 4a–e and Figure S7) show that Zn^2+^ ions tend to be adsorbed at the top position of the Zn-(002) surface, whereas the exposed dangling CN^-^ sites of the cleaved PBA-(002) are preferred for desolvated Zn^2+^-ion. The corresponding Zn^2+^-ion adsorption energies are −0.60 eV and −4.99 eV for Zn-(002) and PBA-(002) slabs, respectively. The lower binding energy of Zn^2+^ confirms that the PBA layer has stronger zincophilicity than bare Zn, which helps to promote the Zn^2+^ desolvation process at the anode/electrolyte interface, thereby inhibiting HER and enhancing the homogeneous Zn deposition. We also computed the binding energy between the H_2_O molecule and the PBA-(002) slab, and it is clear that water is more easily trapped at the PBA surface (−1.03 eV) than the bare Zn (−0.29 eV). The strong interactions between water and the PBA surface may curb the further percolation of water molecules and permit the Zn^2+^-ion transportation through open tunnels, thus guiding the ordered deposition of Zn. The mechanism can be illustrated in Figure 4f; the depicted Zn^2+^-ion diffusion channels in the open framework of PBA could provide homogeneous nanochannels for uniform Zn^2+^-ion diffusion, whereas the coordinated water molecules were excluded from entering into the framework. The PBA coating can also effectively increase the ion mobility rate and accelerate the reaction kinetics owing to its strong zincophilic property. In summary, the PBA layer with strong Zn affinity, 3D open channels, and rich zincophilic sites significantly enhances Zn electrode performance through directing ordered Zn^2+^-ion flux, accelerating ion migration, facilitating uniform nucleation, and eventually enabling stable dendrite-free Zn metal plating/stripping.

### 2.4. Full Cell Examination

**Figure 5:**
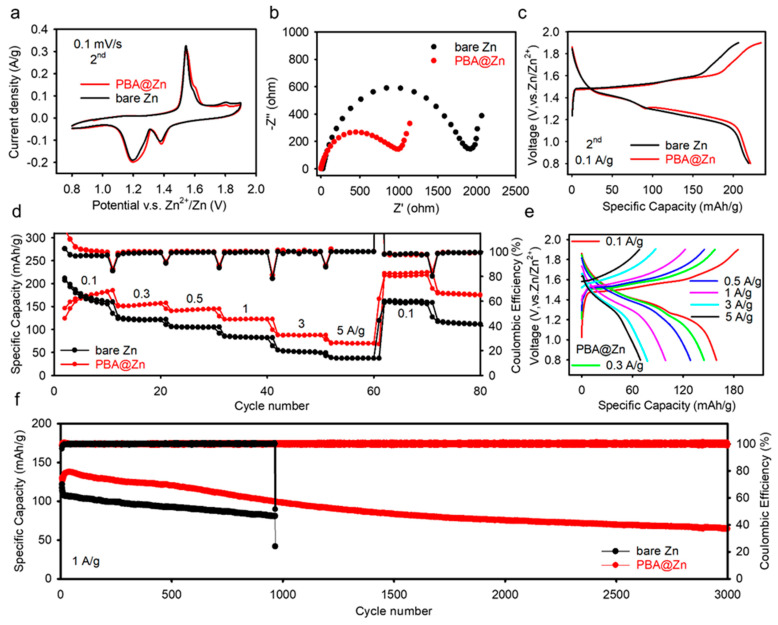
(**a**) CV profiles of the Zn/α-MnO_2_ cells and PBA@Zn/α-MnO_2_ cells, (**b**) Electrochemical impedance spectroscopy (EIS) data of Zn/α-MnO_2_ cells and PBA@Zn/α-MnO_2_ cells, (**c**) Charge−discharge curves of the Zn/α-MnO_2_ cells and PBA@Zn/α-MnO_2_ cells, (**d**) Rate performance of the Zn/α-MnO_2_ cells and PBA@Zn/α-MnO_2_ cells, (**e**) Charge-discharge profile of the PBA@Zn/α-MnO_2_ cells at different current densities, (**f**) Cycling performance of Zn/α-MnO_2_ and PBA@Zn/α-MnO_2_ cells at 1 A g^−1^.

Apart from asymmetric and symmetric cell tests, full batteries using the α-MnO_2_ as the cathode were assembled to estimate the effect of the coating on the full cell. As shown in Figure S8, the peaks of the XRD are consistent with the standard α−MnO_2_ (PDF# 72−1982), and the morphology of scattered accumulated nanorods can be observed in the SEM images. The EDS results show that Mn and O elements are uniformly distributed in the cathode material. In Figure 5a, both CV curves of the full cells with and without the PBA coating have two pairs of peaks, suggesting that the PBA layer does not impact the electrochemical behaviors of the full batteries. Additionally, the almost identical voltage platforms observed in the second-cycle galvanostatic charge-discharge curves of Zn/α-MnO_2_ and PBA@Zn/α-MnO_2_ batteries indicate that the layer has no effects on the redox reactions (Figure 5e). The rate performance of the two electrochemical full cells is presented in Figure 5d. The PBA@Zn/α-MnO_2_ cell displays specific capacities of 167.0, 154.0, 142.9, 122.3, 87.3, and 69.6 mAh g^−1^ at 0.1, 0.3, 0.5, 1.0, 3.0, and 5.0 A g^−1^, respectively. Due to the presence of the PBA membrane, the charge transfer resistance is reduced, and the battery rate performance is improved (Figure 5b). It is noted that the α−MnO_2_ has an activation process that leads to an increase in initial specific capacities. The long lifespan of 3000 cycles, high stability, and high Coulombic efficiency are enabled by the successful suppression of Zn dendrite growth and side reactions by the protective PBA interphase layer. These results highlight that the building of a Zn^2+^-ion-conductive and water-blocked PBA interphase is an effective approach to constructing a dendrite-suppressed Zn anode.

## 3. Materials and Methods

### 3.1. Materials

All chemicals and reagents like Zn foil (0.15 mm thick, Sinopharm Chemical Reagent Co., Ltd., Shanghai, China), K_3_(Fe(CN)_6_) (AR, Aladdin Chemical Reagent Co., Ltd., Shanghai, China), ZnSO_4_·7H_2_O (AR, Sinopharm Chemical Reagent Co., Ltd., Shanghai, China), polyvinylidene difluoride (PVDF, 99%, AR, Aladdin Chemical Reagent Co., Ltd., Shanghai, China), Super-P carbon black (99.8%, Hefei KeJing Materials Technology Co., Ltd., Hefei, China), KMnO_4_ (AR, 99.5%, Sinopharm Chemical Reagent Co., Ltd., Shanghai, China), and HCl (AR, 36.0–38.0%, Sinopharm Chemical Reagent Co., Ltd., Shanghai, China) were purchased and used directly without further processing.

### 3.2. Synthesis of the PBA Coating

First, 10 mM ZnSO_4_·7H_2_O and 10 mM K_3_(Fe(CN)_6_) were dissolved in a beaker containing 100 mL of deionized water separately. Then two homogeneous solutions were obtained after vigorous agitation. The two solutions were mixed and stirred for 15 min, and then left to stand in the beaker. The clean Zn flake was placed in the mixture for reacting 10 min, after which the PBA@Zn could be obtained and finally washed with deionized water.

### 3.3. Synthesis of α-MnO_2_ Cathode Material by Hydrothermal Reactions

5 mM potassium permanganate (KMnO_4_) was dissolved into 24 mL of 1M HCL in a flask. Then, deionized water was added to 70 mL, and the resulting mixed solution was obtained after stirring for 30 min. The mixed solution was transferred to a 100 mL autoclave and reacted at 140 °C for 18 h, and then cooled to room temperature naturally. The reaction kettle was removed, and the precipitate was obtained by centrifugation, and then the samples were washed several times by centrifugation with deionized water and ethanol, and the washed samples were transferred to a vacuum oven and dried at 80 °C for 12 h to obtain α-MnO_2_ nanorods [39].

### 3.4. Synthesis of the α-MnO_2_ Composite Electrode

The slurry was obtained by mixing α-MnO_2_ powder with Super-P carbon black and PVDF with a mass ratio of 70:20:10 in NMP. The prepared slurry was poured on a carbon cloth and dried at 80 °C for 12 h to obtain a composite α-MnO_2_ electrode.

### 3.5. Electrochemical Evaluation

CR2032-type coin cells were assembled under the ambient environment. NEWARE battery testing systems (Neware Technology Co., Ltd., Shenzhen, China) were used to conduct battery performance tests for cycle stability. Bio-Logic (SP-300, Bio-Logic Science Instruments Co., Ltd., Seyssinet-Pariset, France) and an electrochemical workstation (CHI 660e, Shanghai Chenhua Instrument Co., Ltd., Shanghai, China) were employed to analyze other electrochemical behaviors.

### 3.6. Material Characterization

X-ray diffraction (Ultima IV, Rigaku Corporation, Japan) analysis was used to examine the crystal phases of the samples at a scanning rate of 10 min^−1^ in the 2θ ranges from 5° to 80°, with an operating voltage and current of 40 kV and 40 mA, respectively. A scanning electron microscope (Pheom LE, Thermo Fisher Scientific, Nn Bleiswijk, The Netherlands) was applied to investigate the microstructure and morphology of the as-synthesized samples. Fourier-transform infrared spectroscopy (FTIR) (Nicolet iS 5, Thermo Fisher Scientific, Nn Bleiswijk, The Netherlands) was used for the functional group analysis.

### 3.7. Details of Density Functional Theory (DFT) Calculation

The hexagonal P6_3_/mmc-Zn (space group # 194) and trigonal Zn_3_[Fe(CN)_6_]_2_ with the R-3c symmetry (space group # 167) were employed in the first-principles calculations. The Zn-(002) crystal plane was cleaved, and the constructed slab model comprises five layers of Zn atoms and a vacuum layer of 15 Å. A supercell of 4 × 4 × 1 was generated to eliminate the artificial interactions between the adsorbates of neighboring periodic images. As for Zn_3_[Fe(CN)_6_]_2_, we created the (002) surface with a chemical formula Zn_9_[Fe(CN)_6_]_6_, and the adjacent PBA layers stacked in [001] direction were separated at least 15 Å. Those structures were subjected to relaxations using the density functional theory (DFT) implemented in the Vienna Ab initio Simulation Package (VASP) [40]. Atomic coordinates were allowed to change during optimization, whereas the edges and shapes of surface models were kept fixed. The H, O, C, N, Zn, and Fe with outer electron configurations of 1s^1^, 2s^2^2p^4^, 2s^2^2p^2^, 2s^2^2p^3^, 3d^10^4s^2^, and 3p^6^3d^6^4s^2^, respectively, were treated as valence electrons. We adopted the projector-augmented wave (PAW) [41] pseudopotentials to describe the ion cores, and the Perdew–Burke–Ernzerhof (PBE) functional of generalized gradient approximation (GGA) [42] to evaluate the exchange-correlation interactions of the valence electrons. The spin-polarized calculations were carried out with a cutoff energy of 400 eV, and respective Γ-centered Monkhorst–Pack 3 × 3 × 1 and 2 × 2 × 1 k-point meshes were used to integrate the Brillouin zones (BZs) of Zn-(002) and PBA-(002), respectively. The dispersion interactions were taken into consideration by applying the D3 method proposed by Grimme [43]. To correct the on-site Coulombic repulsions of the localized Fe 3d-electron, a Hubbard U value of 5.3 eV [44] was used. The convergence criteria for the forces exerted on each atom and total energy change between each electronic iteration were 0.05 eV/Å and 1 × 10^−5^ eV, respectively. The binding energy is defined as E_binding_ = E_slab + adsorbate_ − E_slab_ − E_adsorbate_. Three adsorption sites including hollow, top, and bridge were considered for the Zn metal surface, and only one position was included for the PBA slab in terms of the complexity of the multiple elements model. All the binding energy calculations were automated using the Python Materials Genomics (Pymatgen) module [45].

## 4. Conclusions

We demonstrated that the porous PBA layer not only effectively inhibited the generation of hydrogen and the corrosion occurring at the Zn anode but also regulated the Zn^2+^-ion flux at the electrolyte/anode interface. Moreover, the rapid charge transfer and the redistribution of Zn^2+^-ion flux brought by the zincophilic interphase formed during the in-situ growth provide significant advantages for achieving uniform and stable Zn deposition. Meanwhile, the coating can effectively inhibit the corrosion occurring at the zinc anode side. Thanks to the PBA covering, the Zn/Zn symmetrical cell exhibited a stable durability over 860 h at a high current density of 5 mA cm^−2^, and the reversibility and Coulombic efficiency of the Zn/Cu cell were improved significantly. The practical feasibility of using the PBA layer was validated in the PBA@Zn/α-MnO_2_ full cell, which delivered a long lifespan of 3000 cycles at 1 A g^−1^. In summary, the PBA layer could notably enhance the cycling stability of Zn anode, and it holds promise for application in next-generation high-performance ZIBs.

## Data Availability

The data presented in this study are available in article and Supplementary Material.

## Supplementary Materials

The following supporting information can be downloaded at: https://www.mdpi.com/article/10.3390/molecules29071501/s1, Figure S1: Optical and SEM images of (a) bare Zn and Zn foils after reacting for (b) 1 min, (c) 5 min, (d) 10 min. Figure S2: EDS mapping of the Zn plate after coating for 10 min. Figure S3: FTIR spectrum of PBA layer. Figure S4: The SEM images of (a) bare Zn, (b) PBA@Zn after cycle for 50 h at 5 mA cm^−2^, 2 mAh cm^−2^. Figure S5: The cross-sectional SEM images of bare Zn and PBA@Zn. Figure S6: The XRD image of bare Zn and PBA@Zn after cycle for 50 h at 5 mA cm^−2^, 2 mAh cm^−2^. Figure S7: Optimized structural models of (a) PBA-(002), and (b) Zn-(002). Figure S8: XRD, SEM images, and EDS mapping of α-MnO_2_. Figure S9: CV profiles of the Zn/α-MnO_2_ cells with (a) bare Zn, (b) PBA@Zn anodes. Figure S10: Charge-discharge curves at 0.1 A g^−1^ (a) bare Zn/α-MnO_2_, (b) PBA@Zn/α-MnO_2_. Figure S11: Cycling performance and Coulombic efficiency of bare Zn/α-MnO_2_ and PBA@Zn/α-MnO_2_ at (a) 0.1 A g^−1^, (b) 0.5 A g^−1^.
