# Supplementary material for: Achieving Long-Cycle-Life Zinc-Ion Batteries through a Zincophilic Prussian Blue Analogue Interphase
Source: Molecules. 2024 Mar 27;29(7):1501. doi: 10.3390/molecules29071501 (PMC11013475; doi:10.3390/molecules29071501)
Supplement: Supplementary file 1 [file molecules-29-01501-s001.zip › molecules-2913649-supplementary.pdf]

Supporting Information

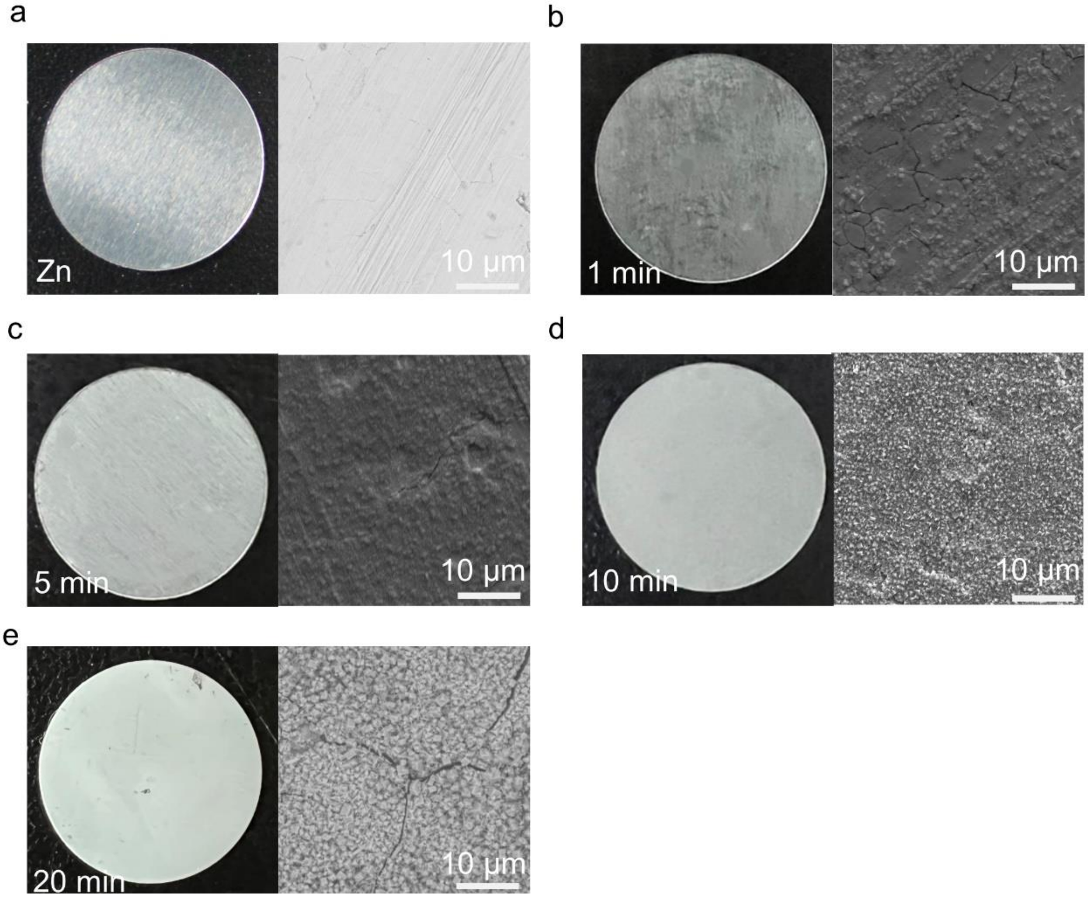

FigureS1. Optical and SEM images of (a) bare Zn, and Zn foils after reacting for (b) 1 min, (c) 5 min, (d) 10 min, (e) 20min.

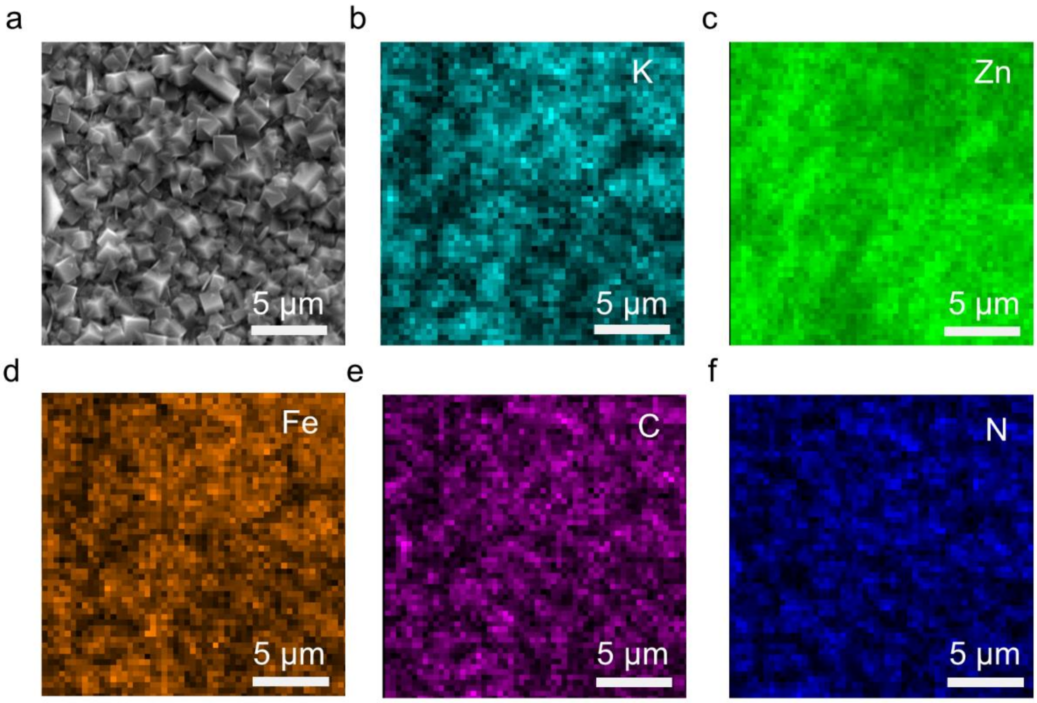

Figure S2. EDS mapping of the Zn plate after coating for 10 min.

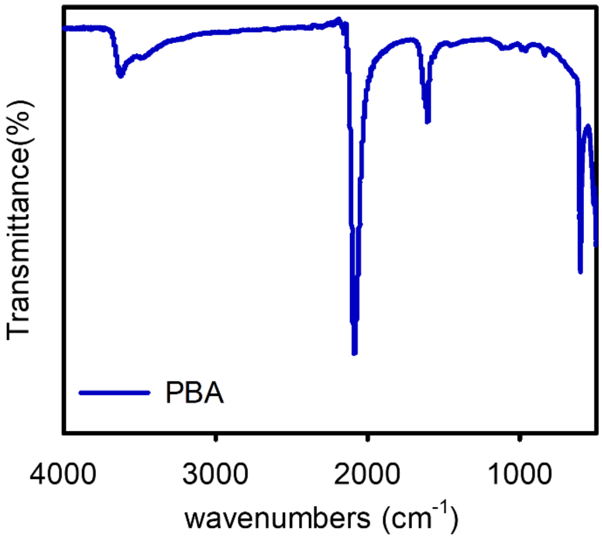

Figure S3. FT-IR spectrum of PBA layer.

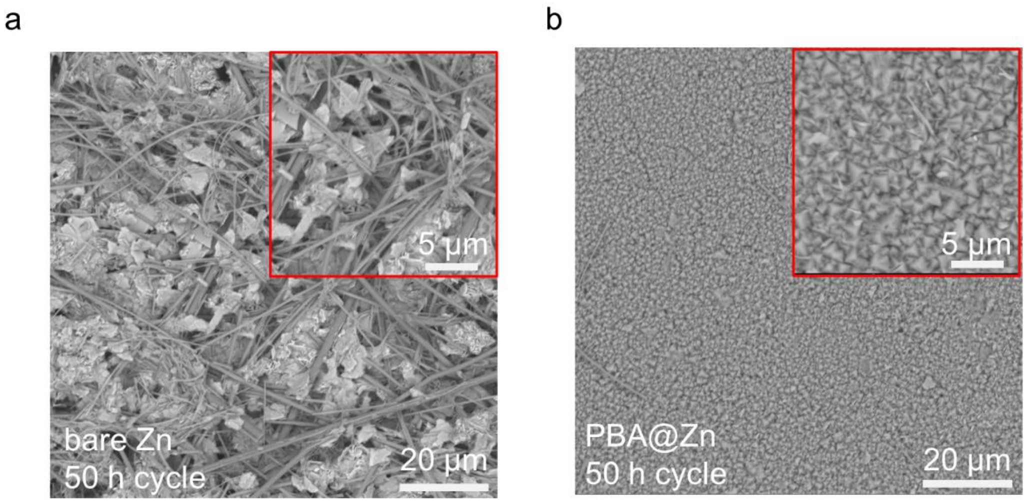

Figure S4. The SEM images of (a) bare Zn, (b) PBA@Zn after cycle for 50 h at 5 mA cm^{-2}, 2 mAh cm^{-2}.

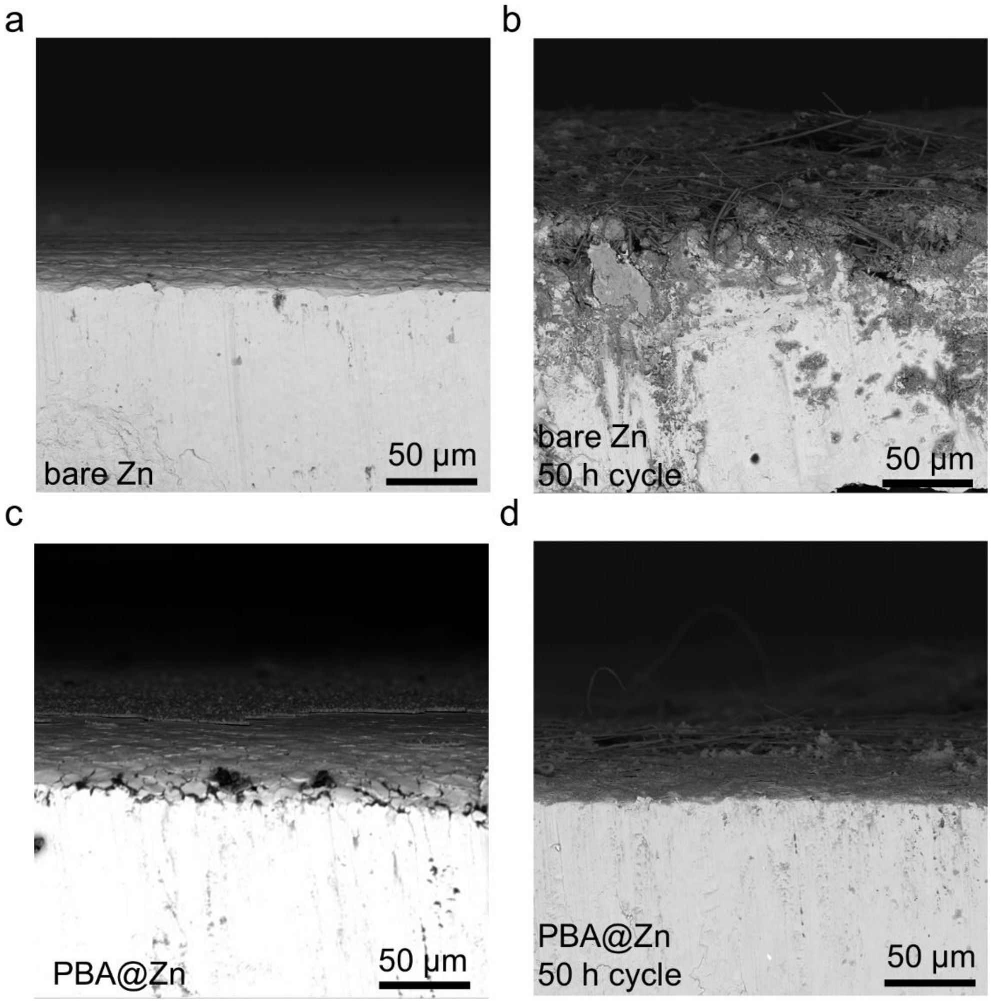

Figure S5. The cross-sectional SEM images of bare Zn and PBA@Zn.

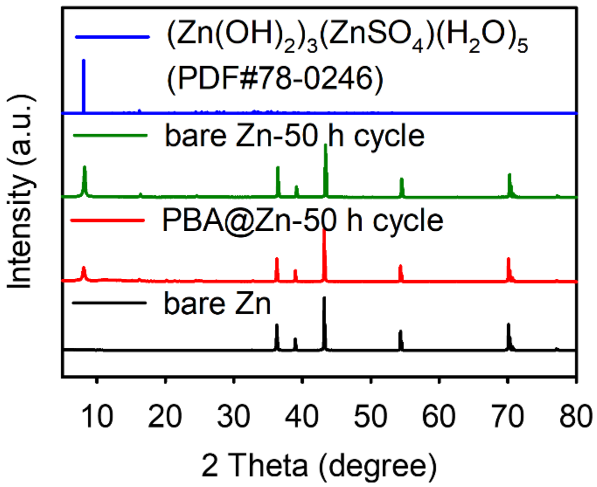

Figure S6. The XRD image of bare Zn and PBA@Zn after cycle for 50 h at 5 mA cm^{-2}, 2 mAh cm^{-2}.

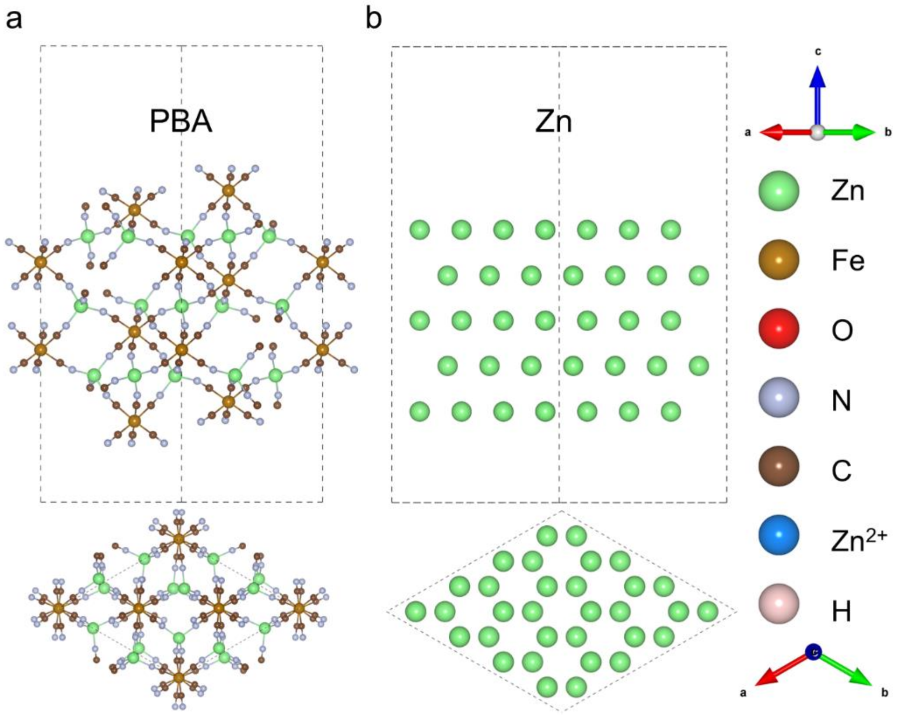

Figure S7. Optimized structural models of (a) PBA-(002), and (b) Zn-(002).

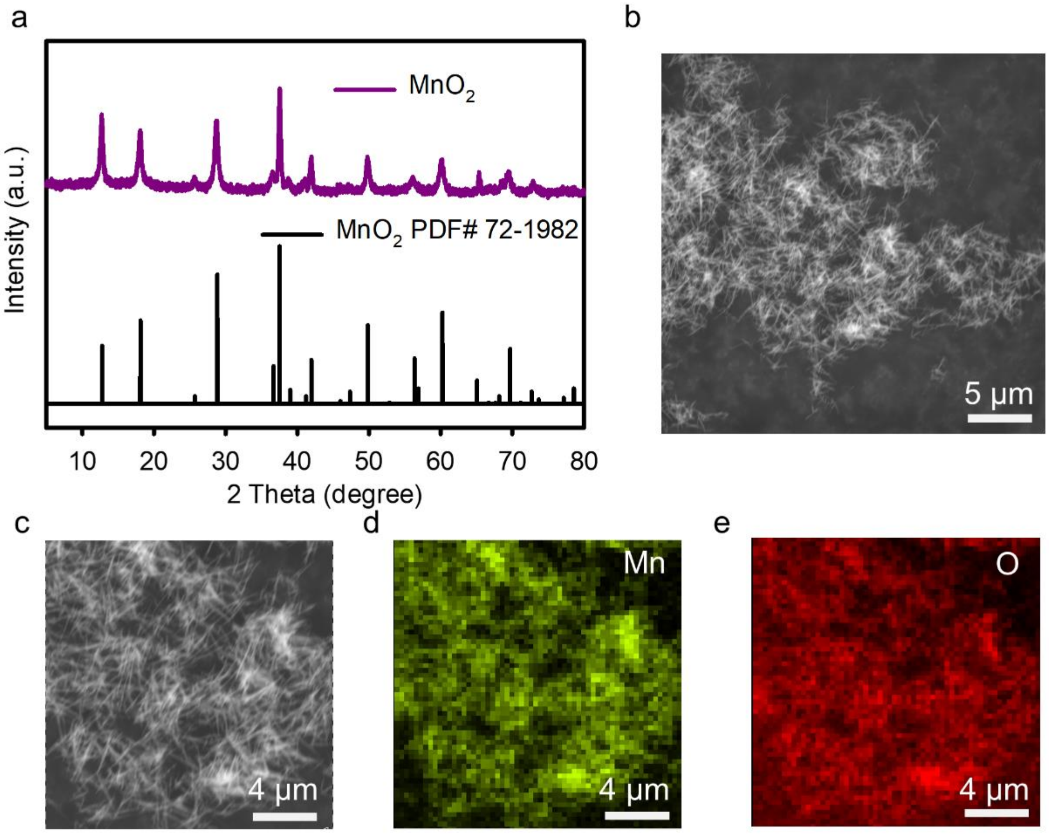

Figure S8. XRD pattern, SEM images, and EDS mapping of α-MnO_2.

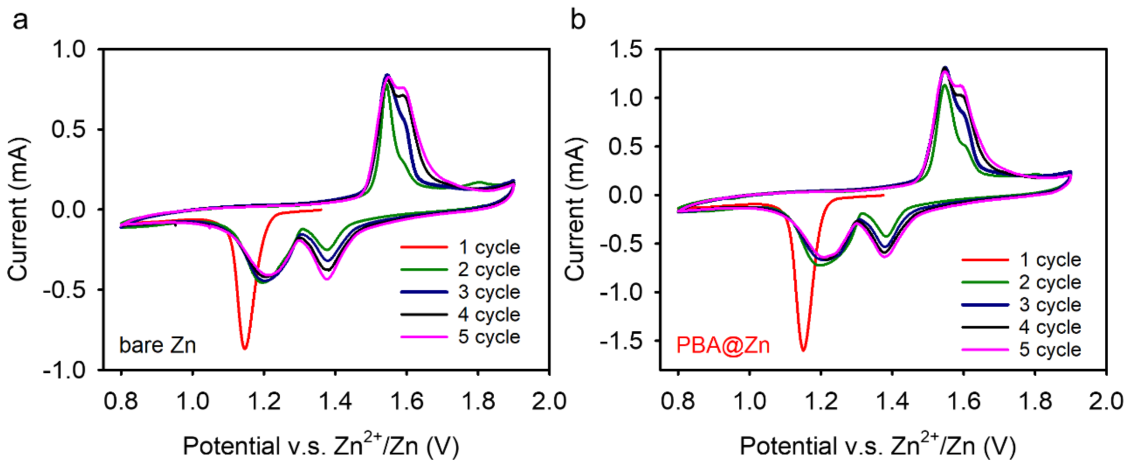

FigureS9. CV profiles of the Zn/α-MnO_2 cells with (a) bare Zn, (b) PBA@Zn anodes.

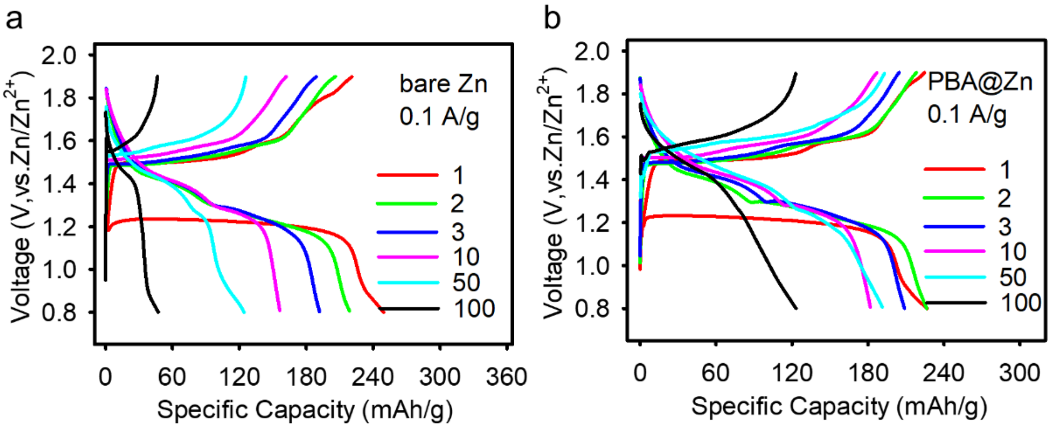

Figure S10. Charge-discharge curves at 0.1 A g^{-1}, (a) bare Zn/α-MnO_2, (b) PBA@Zn/ α-MnO_2.

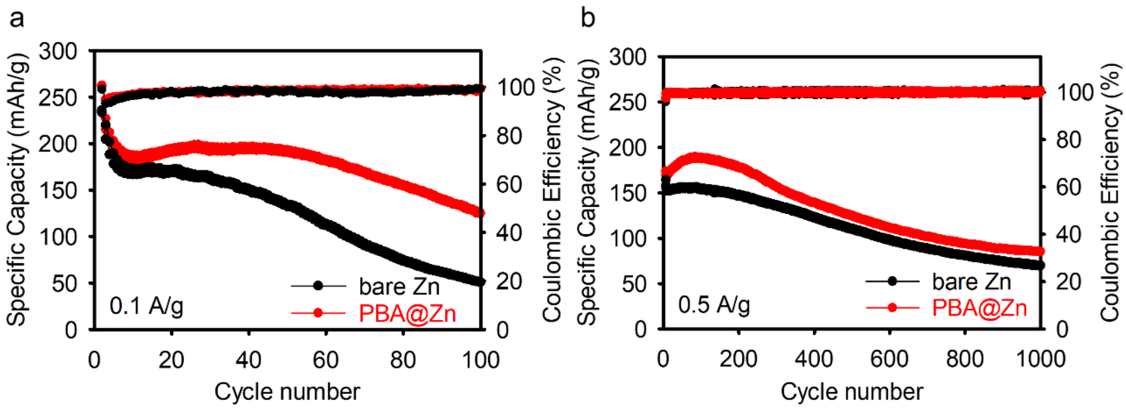

Figure S11: Cycling performance and coulombic efficiency of bare Zn/α-MnO_2 and PBA@Zn/ α-MnO_2 at (a) 0.1 A g^{-1}, (b) 0.5 A g^{-1}.
